# NGS Nominated *CELA1*, *HSPG2*, and *KCNK5* as Candidate Genes for Predisposition to Balkan Endemic Nephropathy

**DOI:** 10.1155/2014/920723

**Published:** 2014-05-15

**Authors:** D. Toncheva, M. Mihailova-Hristova, R. Vazharova, R. Staneva, S. Karachanak, P. Dimitrov, V. Simeonov, S. Ivanov, L. Balabanski, D. Serbezov, M. Malinov, V. Stefanovic, R. Čukuranović, M. Polenakovic, L. Jankovic-Velickovic, V. Djordjevic, T. Jevtovic-Stoimenov, D. Plaseska-Karanfilska, A. Galabov, V. Djonov, I. Dimova

**Affiliations:** ^1^Department of Medical Genetics, Medical University of Sofia, Zdrave Street 2, 1431 Sofia, Bulgaria; ^2^Genomics Laboratory of Malinov Clinic, 1620 Sofia, Bulgaria; ^3^Vratza District Hospital, 66 “Vtori Iuni” Boulevard, 3000 Vratza, Bulgaria; ^4^Faculty of Medicine, University of Nis, Univerzitetski trg 2, 18000 Nis, Serbia; ^5^Faculty of Medicine, University of Skopje, Macedonian Academy of Sciences and Arts, Bul. Krste Misirkov 2, P.O. Box 428, 1000 Skopje, Macedonia; ^6^Institute of Microbiology, Bulgarian Academy of Sciences, 26 Georgi Bonchev Street, 1113 Sofia, Bulgaria; ^7^National Center of Public Health and Analyses, 15 Acad. Ivan Evst. Geshov Boulevard, 1431 Sofia, Bulgaria; ^8^Institute of Anatomy, Bern University, Baltzerstrass 2, 3012 Bern, Switzerland

## Abstract

Balkan endemic nephropathy (BEN) is a familial chronic tubulointerstitial disease with insidious onset and slow progression leading to terminal renal failure. The results of molecular biological investigations propose that BEN is a multifactorial disease with genetic predisposition to environmental risk agents. Exome sequencing of 22 000 genes with Illumina Nextera Exome Enrichment Kit was performed on 22 DNA samples (11 Bulgarian patients and 11 Serbian patients). Software analysis was performed via NextGene, Provean, and PolyPhen. The frequency of all annotated genetic variants with deleterious/damaging effect was compared with those of European populations. Then we focused on nonannotated variants (with no data available about them and not found in healthy Bulgarian controls). There is no statistically significant difference between annotated variants in BEN patients and European populations. From nonannotated variants with more than 40% frequency in both patients' groups, we nominated 3 genes with possible deleterious/damaging variants—*CELA1, HSPG2*, and *KCNK5*. Mutant genes (*CELA1, HSPG2*, and *KCNK5*) in BEN patients encode proteins involved in basement membrane/extracellular matrix and vascular tone, tightly connected to process of angiogenesis. We suggest that an abnormal process of angiogenesis plays a key role in the molecular pathogenesis of BEN.

## 1. Introduction 

Balkan endemic nephropathy (BEN) is a familial chronic tubulointerstitial disease with insidious onset and slow progression to terminal renal failure. It was first described in Serbia and in Bulgaria. The disease affects people living in the alluvial plains along the tributaries of the Danube River in Serbia, Bosnia, Croatia, Bulgaria, and Rumania [1]. Investigations were directed to the epidemiology, etiology, morphology, and treatment of BEN. Results of these studies were reviewed elsewhere [2, 3]. BEN has an onset of between the 40s and 60s, with a long preclinical period. The disease affects both genders with slight female predominance and often leads to terminal kidney failure. About 30–40% of the affected individuals develop uroepithelial tumours of the upper urinary tract [4]. These are mostly papillar carcinomas and are the most common causes of death in BEN patients.

Different chemical elements, organic and nonorganic compounds, viruses, and microorganisms are implicated in BEN development. Heavy metals such as Mg, Mo, Cd, Pb As, and Se can be important for the development of the disease, but there is no clear evidence of their direct toxic effect on the development of the disease. Pathomorphologically BEN has similarities with Chinese herbal nephropathy, which is probably caused by the toxic effect of aristolochic acid, but there is no evidence supporting this theory for BEN. Other possible agents involved in the etiology of BEN are ochratoxin A and some viruses such as picornavirus, polyomavirus, herpes simplex 1 and 2, adenovirus, hepatitis B, cytomegalovirus, and Epstein-Barr virus [5, 6]. For now there is no unchallenged evidence supporting a viral etiopathogenesis of BEN.

BEN is a multifactorial disease with genetic predisposition to environmental risk agents. Familial manifestation of BEN implies a polygenic genetic predisposition [7, 8].

Previous studies have suggested genes located in chromosome band 3q25-3q26, genes for xenobiotic metabolizing enzymes, tumour-suppressor genes, and protooncogenes. The incidence of rapid debrisoquine metabolizers is higher in BEN patients than in healthy controls [9].* CYP2D6 *polymorphic variants predisposing to toxic effect of various chemical agents are suspected in BEN pathogenesis.* LCAT*-deficient individuals have evidence of renaltubular injury and this defect can be involved in BEN [10]. Cytogenetic research on lymphocyte cultures from BEN patients showed that* in vitro *higher folic acid inducedchromosomal fragility and more frequent spontaneous chromosomal aberrations. Some of the unstable regions contain oncogenes—1p36-*C-SRC*, 3p25-*RAF1*, 3q27*-FIM3*, 6q23-*MYB*, 1p13-*NRAS,* and 6p11-*KRAS1P* [11, 12].

Other studies show that environmental factors are very important and can influence genome function without changing the DNA sequence itself. The concept of epigenetics was suggested. The major epigenetic modifications include DNA methylation, histone modifications, and miRNA interference [13]. Epigenetic changes over time display familial clustering [14] and could be implicated in transmitting a “predisposition” over generations. Epigenetic modifications being heritable and adaptable at the same time may prove to make a significant contribution to BEN development and may be the link between the effect of environmental factors and genetic composition in BEN progression. In a previous study we have investigated the methylation status across the whole genome in different patient groups, based on gender and endemic region, in comparison to healthy controls from nonendemic regions. Differentially methylated regions (DMRs) were determined in BEN patient and controls and the commonly presented DMRs were determined to be the most promising methylation alterations in BEN.* SEC61G, IL17RA, *and* HDAC11* proved to be differently methylated throughout all patient-control pairs [15].

In the present study we aimed to perform exome sequencing of 22 000 genes with the Illumina Nextera Exome Enrichment Kit using NGS technology in order to find specific mutations for BEN.

## 2. Materials and Methods

Twenty-two BEN patients were selected for NGS exome analysis. Informed consent was received from all participants enrolled in the study. We obtained peripheral blood samples from 2 series of patients—11 Bulgarian and 11 Serbian. Clinical assessment was performed according to unified criteria and was applied to all sample cohorts. Genealogical analysis was performed to exclude relatives among all study subjects.

Bulgarian samples were collected by preliminary clinical screening in Vratza endemic regions in Bulgaria in 2003 [16]. All subjects were born of Bulgarian ancestry, born and living in the endemic region. DNA was extracted by standard phenol-chloroform extraction procedure and stored at −80°C. All samples were checked for DNA consistency by 1% gel electrophoresis.

Serbian samples were collected from Serbian endemic regions. DNA was extracted by DNA extraction kit and stored at −80°C. All samples were checked for DNA consistency by 1% gel electrophoresis.

The study was approved by the Serbian Ethics Committee of the University of Nis, School of Medicine, Nis, Serbia, and the Bulgarian Commission of Medical Ethics at the National Center of Hygiene, Medical ecology and Nutrition, Sofia, Bulgaria.

### 2.1. Library Preparation and Enrichment

The workflow in the Nextera Enrichment Sample Preparation Guide (Revision B) by Illumina was followed to prepare the libraries for whole-exome sequencing. A Nextera Exome Enrichment Kit was used. The 22 libraries were distributed in 10 enrichment reactions (“pools”)—in 6 of them DNA from 3 libraries was mixed together, while the other 4 contained DNA from only one. The latter were prepared in order to obtain higher mean coverage for these samples. A different DNA quantity (500 ÷ 1000 ng) was added to form each pool depending on the quantity of the least concentrated sample in the reaction.

### 2.2. Library Quantification

After completing the enrichment procedure, DNA concentration of the 10 sequencing-ready pools was measured using a KAPA Library Quantification Kit. The reactions were run in triplicate on an Illumina qPCR Eco system. The Eco Study software was used to calculate the concentrations of the dilutions and then of the stocks.

### 2.3. Denaturation and Dilution

Pools 5–10 were denatured and diluted according to the Illumina guidelines in Preparing DNA Libraries for Sequencing on the MiSeq (Illumina, San Diego, USA). Due to lower concentrations Pools 1–4 were prepared for sequencing following the corresponding chapter in the TruSeq Custom Amplicon Library Preparation Guide, which uses heat denaturation instead of denaturation with 0.1 N NaOH.

### 2.4. Sequencing

The pools were sequenced on an Illumina MiSeq System using the MiSeq Reagent Kit v2 and a 500-cycle 14-tile flow cell. Only Pool 9 was sequenced using a 300-cycle MiSeq Reagent Micro Kit v2 and a micro 4-tile flow cell. We experimented loading the flow cell at different pool concentrations (10 ÷ 30 pM) to establish what the optimal range is for the most efficient cluster density and hence yield.

### 2.5. Analysis

Analysis of the sequencing data was performed using the Softgenetics NextGene Software (version 2.3.3). The variants found were further analyzed by mutation prediction software Provean and PolyPhen-2 to filter the mutations, which are predicted to be deleterious/damaging. We searched for repeated mutations that are unique or that have significantly higher frequencies in BEN patients as compared to individuals from European populations. We focused on nonannotated variants, predicted as possibly deleterious/damaging by software analysis. We selected mutations based on the following criteria: (i) no data about variants; (ii) no incidence in healthy Bulgarian controls and in European populations; (iii) similar incidence in both Bulgarian and Serbian patients' groups; (iv) mutation frequency of more than 40% in BEN patients.

## 3. Results

Twenty-two Bulgarian and Serbian BEN patients were analyzed by NGS exome sequencing for 22 000 genes. Using the Softgenetics NextGene Software (version 2.3.3) the sequencing data were analyzed. Mutation prediction was performed by software Provean and PolyPhen-2.

We discovered in total 3666 missense variants with possible deleterious/damaging effect in our patients' groups. Among them, 1849 (50%) were not annotated.

In total, 980 nonsense and frameshift variants were detected in our study. Among them, 541 (55%) variants have not been annotated in human genome data base of genetic variations or no information about their frequency was available.

Among the annotated variants with possible deleterious/damaging effect we did not find statistically significant difference in the frequency between BEN patients and European populations. From nonannotated variants, we selected the variants with frequency of more than 40% in BEN patients—*HSPG2*,* CELA1*, and* KCNK5* (Table 1). These mutations, alone or in combination, occur in 77% of BEN patients.

**Figure 1:**
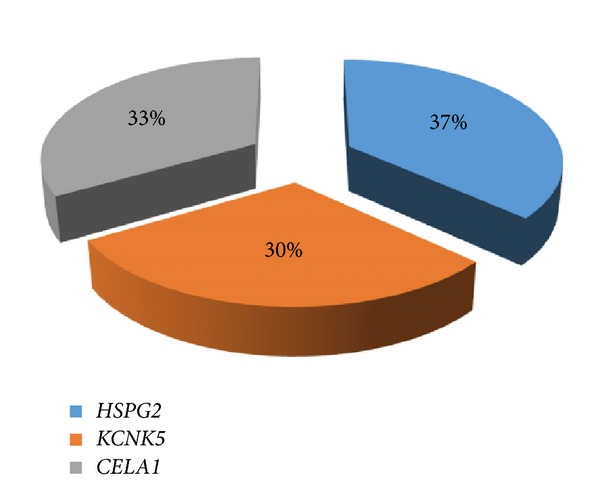
Nominated candidate genes, associated with BEN.

**Figure 2:**
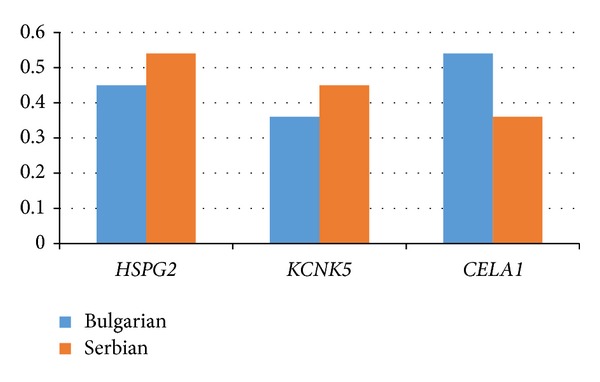
Frequency of nominated mutant genes in Bulgarian and Serbian BEN patients.

The probable contribution of each of the nominated genes to the pathogenesis of BEN (according to their mutation frequency) is represented in Figure 1. The frequencies of each of the nominated mutant genes for Bulgarian and Serbian BEN patients are given in Figure 2. All selected variants occur with similar frequency in the Bulgarian and Serbian groups of BEN patients. The nominated variants are classified according to their function—genes involved in the constitution of basement membrane/extracellular matrix (ECM) (*HSPG2* and* CELA1*) and gene for renal potassium transport and membrane potential (*KCNK5*).

**Figure 3:**
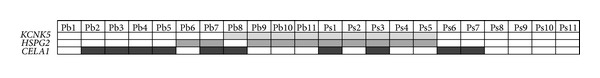
Combination of the three mutant genes found in all BEN patients. Pb: Bulgarian patients; Ps: Serbian patients.

Figure 3 represents the distribution of the three mutant genes in all BEN patients.* KCNK5* variant c.1397A>C occurs only in combination with one or two of the other variants (*HSPG2* c.5239A>C and* CELA1* c.9_10delC), while* CELA1* variant is present as a single aberration in 6 out of 10 positive cases. In 5 of BEN patients none of these variants were found. This is probably due to the presence of other rare mutations, which did not pass the threshold criteria in our study (presented in more than 40% of the patients).

## 4. Discussion

Balkan endemic nephropathy has been traditionally described as an end-stage kidney disease, characterized by bilaterally and symmetrically contracted kidneys of a very small size and reduced weight, coinciding with multiple upper urinary tract tumors in 8–48% of cases [17]. Basic histomorphological changes include tubular atrophy and interstitial fibrosis with sclerosed glomeruli usually of collapsing obsolescence and sclerotic changes in blood vessels. The hypothesis for BEN multifactorial pathogenesis [8] proposes that inherited genetic defects predispose the kidney to damage after exposure to different agents—toxic, immune, or infective. The familial clustering of the disease has prompted genetic investigations [12, 18, 19].

This is the first analysis by NGS sequencing in BEN patients aimed at discovering mutations associated with the disease. Despite intensive molecular studies in BEN, the etiopathogenesis of the disease is still not elucidated and there is no biomarker for disease predisposition. Most of the molecular studies so far have focused on single genes/polymorphisms and very limited information has been provided. Because gene-by-gene analysis by Sanger sequencing is too laborious and expensive, genetic testing has been the exception until recently. Now, next-generation sequencing (NGS) allows for simultaneous and efficient analysis of all known genes for a given trait. Here we applied exome sequencing (comprised of 22 000 genes) in 22 BEN patients in order to detect the most prominent genetic variants with highly probable pathological effect. Recently Poon et al. have applied whole-genome and exome sequencing for analysis of Aristolochic acid- (AA-) associated upper urinary tract urothelial cell carcinoma (UTUC) [20]. AA is a carcinogen that can cause nephrotoxicity as well. Authors observed a high frequency of somatic mutations in chromatin modifiers, particularly KDM6A, in AA-UTUC, demonstrated the sufficiency of AA to induce renal dysplasia in mice, and reproduced the AA mutational signature in experimentally treated human renal tubular cells. Our study was looking for germ-line mutations predisposing to another specific nephropathy-Balkan endemic nephropathy.

Three genes (*HSPG2, CELA1,* and* KCNK5*) were nominated as related to the pathogenesis of BEN based on their mutation frequency, their similar incidence in both Bulgarian and Serbian patients' groups, lack of information about the established variants in European population, and nonincidence in healthy Bulgarians. Analysis of their function sheds light on the possible pathophysiology in BEN, which we discuss here.

The first two genes* HSPG2* and* CELA1* are evidently involved in the process of angiogenesis. The gene* KCNK5* encodes a protein for potassium channel, which could also be involved in vascular disease and complications.

The number of patients requiring renal replacement therapy due to end-stage renal disease (ESRD) is increasing worldwide [21]. The prevalence of chronic kidney disease (CKD) and the importance of CKD as a risk factor in development of ESRD have been confirmed. In recent years, the involvement of angiogenesis-related factors in the progression of CKD has been studied, and the potential therapeutic effects on CKD of modulating these factors have been identified [22]. A number of angiogenic growth factors are involved in the development of the kidney and in the maintenance of glomerular structures and the glomerular filtration barrier function in adults.

Our study revealed significant candidate gene—*HSPG2*, which encodes perlecan protein, a major component of basement membranes, where it is involved in the stabilization of other molecules important for glomerular permeability to macromolecules and for cell adhesion [23, 24]. It binds to and crosslinks many extracellular matrix components and cell-surface molecules [25–29]. It has been shown that this protein interacts with laminin, prolargin, collagen type IV, tenascin-C, FGFBP1, FBLN2, FGF7, transthyretin, and so forth, and plays essential roles in multiple biological activities. Cukuranovic et al. have intensively studied the pathological changes in the kidneys of BEN patients and presented evidence that renal vascular changes occur early in Balkan nephropathy [30]. They detected by IHC a marked overexpression of laminin in renal interstitial capillaries. The pattern of laminin staining in glomeruli corresponded to focal and segmental glomerular sclerosis present in the advanced stages of Balkan nephropathy. Later stages were characterized by an intensive expression of laminin in atrophic tubules, much more in proximal than in distal ones. The coexpression of vimentin and cytokeratin in proximal tubular cells was also demonstrated. The changes described, particularly those taking place at the level of interstitium, bear the key responsibility for BEN progression.

*CELA1*, the second nominated gene, is also involved in the process of angiogenesis [31]. The gene encodes elastase-1, which degrades elastin in the vascular matrix. Tumor angiogenesis, chicken angiogenesis, and mesenteric angiogenesis data suggest that elastin and elastin degradation products play a key role in vascular morphogenesis. CELA1 was expressed in vascular cells in the embryonic lung and in a fetal mesenchymal cell line with angiogenic properties [32]. Degraded elastin causes deposition of hydroxyapatite-like mineral and osteogenic transformation of vascular smooth muscle cells (as they lose the specific *α*-SMA), resulting in vascular calcification [33]. In contrast, collagen-1 levels in areas of calcification are increasing [34]. Changes in both *α*-SMA and elastin inversely correlate with the hemodynamic parameters such as pulse wave velocity (PWV) and lead to media remodeling. This was associated with the increased arterial stiffness observed in CKD rats with vascular calcification. Dysregulation of normal anticalcification factors and elastin degradation represent a pattern of vascular injury existing in patients with end-stage renal diseases [35].

The third gene nominated in our study was the potassium channel gene* KCNK5*. Potassium channels in the kidney play an essential role in controlling and maintaining plasma potassium levels in the normal range, as well as exerting very different functions such as cell volume control, membrane potential stabilization and excitability, or regulation of hormone or ion secretion [36–39]. In addition, potassium ion (K^+^) channel activity is a major regulator of vascular muscle cell membrane potential and is therefore an important determinant of vascular tone. There is growing evidence that the function of several types of vascular K^+^ channels is altered during major cardiovascular diseases, such as chronic hypertension, diabetes, and atherosclerosis [40]. Defects in potassium channels cause abnormal vasodilation responses reflecting a gradual deterioration of vascular mechanisms during the progression of diabetic nephropathy [41]. Enhanced dilator responses and basal activation of K^+^ channels may occur in the renal circulation early during diabetes. An increased K^+^ channel activity may therefore reflect a very high metabolic state of vascular smooth muscle cells [42].

## 5. Conclusion Hypothesis for the Pathogenic Mechanism in BEN

**Figure 4:**
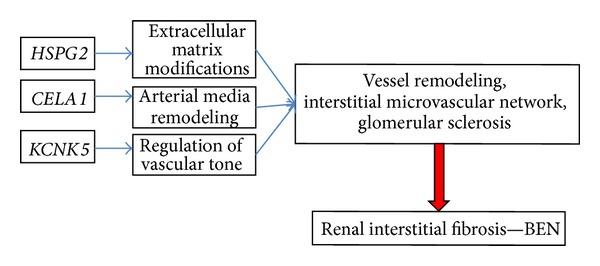
Proposed model for molecular pathogenesis of BEN kidney pathology.

The molecular mechanisms leading to interstitial fibrosis and chronic kidney disease are complex and are probably related to the primary processes leading to renal injury. As blood vessels nourish all the tissues and organs in the body, abnormal formation and remodeling of blood vessels probably contribute to the pathogenesis of renal fibrosis. Neoangiogenesis is a complex process of recruitment, migration, proliferation, and apoptosis of stem/progenitor cells, endothelial cells, vascular smooth muscle cells, and other mural cells. The extracellular matrix plays important roles in vessel development via providing a supportive matrix scaffold and growth factors for cells. Close interactions between vascular cells and their ECM is crucial in blood vessel formation and remodeling. Our results suggest three new genes for predisposition to BEN pathology, related to angiogenic alterations. We hypothesize that mutations in* HSPG2*,* CELA1,* and* KCNK5* participate in extracellular matrix modifications, arterial media remodeling, and regulation of vascular tone, all these events leading to interstitial vessel remodeling, connected to renal interstitial fibrosis in BEN (Figure 4). Further elucidation of molecular pathogenesis of kidney fibrosis could lead to the development of a new target therapy of BEN by targeting specific angiogenic factors. Our results provide a basis for further investigations of the role of the nominated genes in BEN kidney pathology, including screening for variants in the nominated genes in a larger cohort of patients. Herein we suggest a possible mechanism between the three candidate genes and BEN.

The main pathological characteristic of BEN kidney is interstitial fibrosis. Abnormal angiogenesis and vascular remodeling probably contribute to pathogenesis of renal fibrosis. Interactions between endothelial cells, vascular smooth muscle cells and progenitor cells with an extracellular matrix (ECM) play an important role in these processes. Scattered glomeruli showing an obvious segmental or global thickening of the capillary walls with a double outline of the glomerular basement membrane were found in early BEN patients.

Interstitial sclerosis could result from the overproduction of extracellular matrix by injured proximal tubular epithelium and interstitial capillary endothelial cells—this could be the pathogenic role of mutated* HSPG2* gene. The increase of the cortical interstitial volume results in resistance of the postglomerular capillary network with impairment of the glomerular flow [43]. This impairment leads to chronic rise in hydrostatic pressure. The increase of the cortical interstitium additionally leads to an increase in the length of diffusion between the tubules and the intertubular and peritubular capillaries. This increase in the length of diffusion subsequently results in the atrophy of the tubules, reduction of reabsorbtion, and therefore impairment of the effective filtration pressure. The haemodynamic changes inversely correlate with *α*-SMA and elastin in vessels and here could be the additional pathogenic role of mutated* CELA1* gene. The mutated* KCNK5* gene is probably a factor stimulating the haemodynamics-driven vascular remodeling.

## Figures and Tables

**Table 1:** Nonannotated variants with frequency of more than 40% in BEN patients, discovered in our study.

Number of patients	Gene	Chr	Position	Exon	Mutation	Aminoacid change	Protein
11	*HSPG2 *	1	22186113	43	c.5239A>C	p.Thr1747Pro	Heparan sulfate proteoglycan 2
10	*CELA1 *	12	51740414	2	c.9_10delC	p.Leu4Phefs	Chymotrypsin-like elastase family, member 1
9	*KCNK5 *	6	39162513	4	c.1397A>C	p.Thr108Pro	Potassium channel, subfamily K, member 5
